# Governance frameworks for COVID-19 research ethics review and oversight in Latin America: an exploratory study

**DOI:** 10.1186/s12910-021-00715-2

**Published:** 2021-11-06

**Authors:** Ana Palmero, Sarah Carracedo, Noelia Cabrera, Alahí Bianchini

**Affiliations:** 1 Directorate of Research for Health, Ministry of Health, 1925 9 de Julio Av, 1091 Buenos Aires, Argentina; 2 Pontifical Catholic University of Peru, Lima, Peru; 3 Center for the Study of State and Society, Buenos Aires, Argentina; 4 School of Law, University of Buenos Aires, Buenos Aires, Argentina

**Keywords:** Research, Ethics, COVID-19, Pandemic, Ethics review, Epidemic, Preparedness

## Abstract

**Background:**

Research has been an essential part of the COVID-19 pandemic response, including in Latin American (LA) countries. However, implementing research in emergency settings poses the challenge of producing valuable knowledge rapidly while upholding research ethical standards. Research ethics committees (RECs) therefore must conduct timely and rigorous ethics reviews and oversight of COVID-19 research. In the LA region, there is limited knowledge on how countries have responded to this need. To address this gap, the objective of our project is to explore if LA countries developed policies to streamline ethics review and oversight of research in response to the pandemic while ensuring its adherence to ethical standards, and to analyze to what extent these governance frameworks are in accordance with international guidance.

**Methods:**

We conducted a descriptive and exploratory study assessing the COVID-19 research ethics governance frameworks of 19 LA countries, considering 4 dimensions based on international COVID-19 ethics guidance documents: (i) ethics review organizational model adopted, (ii) measures to coordinate between RECs and other research stakeholders, (iii) operational guidance for RECs, and (iv) key ethical issues for review and oversight of COVID-19 research.

**Results:**

10 out of 19 LA countries have some policy to streamline ethics review of COVID-19 research. Of these countries only 6 issued comprehensive documents following international guidance that contemplate strategies with recommendations for concrete actions for a timely and rigorous review.

**Conclusion:**

LA countries adopted partial strategies and operational guidance that may demonstrate a lack of a comprehensive view of research ethics for the review and oversight of COVID-19 research. Continuing efforts should be directed to strengthen LA countries' research capacity to respond timely and ethically to future health emergencies. Past lessons and the ones from this pandemic should be the basis to develop international standards and operational guidelines for ethics review and oversight of any research for public health emergencies.

**Supplementary Information:**

The online version contains supplementary material available at 10.1186/s12910-021-00715-2.

## Introduction

The COVID-19 pandemic has severely affected the countries of Latin America (LA) due to social and economic inequalities across their populations, and their deficient health system [1]. By mid-2020, several countries in the region accounted for almost one-third of all COVID-19 deaths worldwide [2]. As in other parts of the world, LA was not prepared to face a health emergency of this magnitude. Among the responses to the COVID-19 pandemic, the conduct of research has been essential. Since the beginning, countries of LA have been actively contributing to the development of vaccines and treatments for COVID-19, despite their limited research capacity and scarce resources [3]. However, implementing research during the pandemic poses the challenge of integrating research into broader outbreak response efforts to produce valuable knowledge rapidly while upholding research ethical standards [4]. In this context, the ethics review and oversight of research become key elements to ensure its social value, the quality of the knowledge generated, the transparency with which it is produced, and the protection of participants.

This issue has long been discussed in previous outbreaks [5–8] and has led to the development of many international guidelines and statements, which highlight the importance of catalyzing ethical research in emergency settings [9–12]. Particularly in LA, the background for this discussion comes from the Zika virus epidemic [13]. These international documents are expected to guide countries to develop research ethics preparedness policies and operational strategies considering their social and cultural context. In this regard, there has been a growing consensus for the implementation of a rapid and robust ethics review system in emergency settings that allows flexibility in research ethics committees (RECs) operating procedures to guarantee a time-sensitive functioning, and increased rigor in their reviews and decisions. For both purposes, several international recommendations for RECs have been identified [14–16].

Since the beginning of the COVID-19 pandemic, the World Health Organization (WHO) and the Pan American Health Organization (PAHO) have emphasized the moral duty to conduct ethical research in response to the pandemic and have developed operational strategies and key ethical issues guidance for ethics review and oversight taking into account the lessons learned from past outbreaks. The goals of these documents are to reduce practical obstacles, save efforts, resources, and time, and ensure a rigorous ethical assessment of COVID-19-related research protocols [17, 18].

Despite the existing international framework for ethics review and oversight in emergency contexts, there is limited knowledge about how LA countries have adapted and formally adopted this ethics guidance to their governance frameworks. To address this gap, our project aimed to identify if LA countries have developed policies and regulations to streamline ethics review and oversight of research in response to the COVID-19 pandemic while ensuring research ethical standards. And if so, to analyze to what extent these governance frameworks are in accordance with international guidance issued by WHO and PAHO.

## Methods

We conducted a descriptive and exploratory study based on a review of the COVID-19 research ethics governance frameworks from different countries of Latin America. The review included Spanish and Portuguese-speaking Latin American countries because of their similar legal culture based on the Civil Law legal system. 19 countries were included: Argentina, Bolivia, Brazil, Chile, Colombia, Cuba, Costa Rica, Dominican Republic, Ecuador, El Salvador, Guatemala, Honduras, Mexico, Nicaragua, Panama, Paraguay, Peru, Uruguay, and Venezuela. Puerto Rico was excluded given that the human research regulations are those of the United States.

We searched for documents, guidelines, recommendations, and any other available instrument on ethics review and oversight of COVID-19 research issued by relevant authorities in national legal databases and governmental websites of national health authorities and national regulatory authorities. To identify documents two categories of search terms were used, (a) research ethics review and (b) COVID 19 pandemic /health emergency, and a combination of related terms. The terms were searched in Spanish and Portuguese. Also, documents were added based on the authors’ knowledge through manual searching. No publication date limit was applied. All documents that addressed the research objectives partially or totally were included. We excluded documents that were too vague or too general to guide research ethics review and oversight of COVID-19 research. When it was needed, government officials working in areas related to health research and research ethics of the countries were contacted by email to validate the information found or for clarification on the content of the documents. We contacted officials of seven countries (Additional file 1). Data collection was conducted between November 1 and December 31, 2020. In total, 21 documents were identified (see Additional file 2). As some countries added new regulations or recommendations during the course of the pandemic, in several cases more than one document was found. In these cases, the whole governance framework of the country was considered as the unit of analysis. To identify the measures that each LA country adopted in the context of the pandemic, it was also necessary to know the status of its research ethics system prior to the COVID-19 pandemic. Therefore, a supplementary search of the human research ethics governance framework of each country was also conducted (Additional file 2).

The documents identified were full-text assessed by three researchers and data was systematized in a novel analysis framework developed by the authors. The dimensions and categories were elaborated based on the guidance for ethics review and oversight of research during the COVID-19 pandemic issued by WHO and PAHO [17–22]. Data collected were organized into 4 dimensions. The first dimension, "Organizational model", aimed to identify the adoption of specific organizational strategies to accelerate COVID-19 research ethics review and oversight. For example, if the country created an ad hoc REC or if it designated a REC of a national governmental entity to specifically review COVID-19-related research. Or, for research projects involving more than one country, if they designated a (sub)regional REC.

The second dimension, "Coordination between stakeholders", was composed of two categories that analyze provisions for the coordination and communication between RECs (for instance, when reviewing multicenter studies), and between RECs and other research stakeholders (e.g., health authorities, national regulatory authorities) in order to avoid duplication of efforts and save valuable time.

The third dimension, "Operational Guidance", aimed to identify guidance for RECs functioning for expedited review of COVID-19 research. Expedited review was defined as a rapid and robust ethics review that allows flexibility in RECs to guarantee a time-sensitive functioning while upholding ethical standards. This dimension is composed of 10 categories:

1. Members’ availability to rapidly review COVID-19 research proposals.
2. Members’ training on emergency research ethics.
3. COVID-19-related experts as members or as independent consultants of RECs.
4. Virtual meetings
5. Reduced quorum when reviewing and deciding about a protocol.
6. Use of electronic means for submissions and communications between members, investigators, authorities, or other research stakeholders.
7. Shorter time frames for the review process (e.g., to organize meetings, send and receive communications, review protocols, and adopt decisions).
8. Advanced review of generic protocols for research in emergencies (protocol models)
9. Decision-making procedures adjusted not to affect the quorum (e.g., staggered deliberations, anticipated decisions by members, etc.)
10. Adjustments of procedures for the ethics oversight of approved studies (e.g., modality, frequency, intensity)

Finally, the fourth dimension, "Key ethical issues for review and oversight" assessed if issues raised by the exceptional context of the pandemic were considered as requirements in projects submitted for ethics review. This dimension was composed of 5 categories:

1. Alternative processes to obtain participants’ informed consent.
2. Processes for the collection and storage of samples and data for future research.
3. Plans to mitigate risks related to the spread of COVID-19 and the strain on healthcare systems.
4. Strategies for community engagement in COVID-19 research.
5. Plans for rapid data sharing.
6. Mechanisms to ensure equal access to research benefits.

## Results

Of the 19 LA countries, 10 countries (53%) issued legal or guidance documents in order to streamline ethics review and oversight of research in response to the COVID-19 pandemic (Table 1). Table 2 presents the strategies adopted by LA countries. In the case of Costa Rica, an organizational mixed strategy was found. On one hand, the National Health Research Council created a COVID-19 ad-hoc committee for specific cases, and the Social Security Administration (CCSS, by its initials in Spanish), which has a leading role in the administration of public health care institutions in the country, issued a manual of procedures to streamline ethics review and oversight of biomedical COVID-19 research for RECs of these institutions.

**Table 1:** Countries and governing instruments issued to streamline COVID-19 research ethics review

Country	Governing instrument	Issuing authority
Argentina	Resolution 908/2020, Ethical and operational guidelines for accelerated ethics review of COVID-19-related human research (May 2020)	Ministry of Health
	Communication ANMAT (May 2020)	ANMAT (NRA*)
Brazil	GUIDELINES FOR CONDUCTING RESEARCH AND REC ACTIVITY DURING THE PANDEMIC CAUSED BY CORONAVIRUS SARS-COV-2	National Research Ethics Commission—CONEP
	Technical Note 23/2020 (July 2020)	ANVISA (NRA)
Chile	Recommendations for scientific ethics committees for research protocols review in the context of the COVID-19 pandemic (June, 2020)	Ministerial Health Research Ethics Commission- CMEIS
Colombia	External Circular 1000–174-20 (July 2020)	INVIMA (NRA)
Costa Rica	Communique 1: Specific considerations for biomedical research in the context of the pandemic (April, 2020)	National Health Research Council—CONIS
	Communique 2: Recommendations for the conduct of biomedical research during the health emergency in Costa Rica (August, 2020)	
	Manual of procedures to streamline scientific and ethics review and oversight of COVID-19-related biomedical research (July, 2020)	Social Security Administration—CCSS
Dominican Republic	Communique, The CONABIOS in times of COVID-19 (April, 2020)	National Council of Bioethics in Health—CONABIOS
Ecuador	Ministerial Agreement No 0003, Regulation for health research during the health emergency (April, 2020) repealed through the Ministerial Agreement No 00,104, Regulation for the approval and the conduct of health research related to COVID-19 (December, 2020)	Ministry of Health
Mexico	Bioethics in the face of the COVID-19 pandemic (March 2020)	National Commission on Bioethics—CONBIOETICA
	Communique No 007 COVID-19 (May 2020)	COFEPRIS (NRA)
	Communication “Extraordinary Measures in relation to Clinical trials during the pandemic” (April 2020)	
Panama	Resolution No 373 (13 April 2020)	Ministry of Health
Peru	Supreme Decree No 014–2020-SA that establishes measures for the adequate conduct of COVID-19 clinical trials in the context of the health emergency (April, 2020)	Ministry of Health
	PNIH Chief Resolution No 096–2020-J-OPE/INS that creates the National Transitory Research Ethics Committee for the ethics review and oversight of COVID-19 clinical trials (April, 2020)	Peruvian National Institute of Health (PNIH)
	PNIH Chief Resolution No 097–2020-J-OPE/INS that approves the procedure for the ethics review of COVID-19 clinical trials (April, 2020)	
	PNIH Chief Resolution No 139–2020-J-OPE/INS that approves guidelines for the conduct of clinical trials during the COVID-19 pandemic (June, 2020)	
	Directorial Resolution No 120–2020-OGITT/INS that approves the NTREC-COVID19 operating procedures (April, 2020)	

*National regulatory authority

**Table 2:** Strategies to facilitate ethics review of COVID-19 research and LA countries that adopted them

Strategy	Categories	Countries	Total
Organization model	Creation of a National Ad-hoc REC for COVID-19 research	Ecuador, Peru	2 (20%)
	Designation of a National REC for COVID-19 research review	Brazil, Dominican Republic, Panama	3 (30%)
	Creation of an Ad-hoc REC for COVID-19 research under specific cases	Costa Rica	1 (10%)
	No changes	Argentina, Chile, Mexico, Colombia	4 (40%)
Coordination between stakeholders	Coordination between RECs	Brazil, Chile	2 (20%)
	Coordination among RECs and other stakeholders (health authorities, NRA)	Colombia (between RECs and NRA)	1 (10%)
Operational guidance	Members’ availability	Argentina, Brazil, Chile, Costa Rica (CCSS), Ecuador, Panama, Peru	7 (70%)
	Emergency ethics training	Argentina	1 (10%)
	Call for COVID-19-related experts	Argentina, Chile, Costa Rica (CCSS), Ecuador, Mexico, Panama, Peru	7 (70%)
	Virtual meetings	Argentina, Brazil, Chile, Costa Rica (CCSS), Dominican Republic, Ecuador, Mexico, Peru	8 (80%)
	Reduced quorum	Argentina, Brazil, Costa Rica (CCSS)	3 (30%)
	Use of electronic means	Argentina, Brazil, Chile, Costa Rica (CCSS), Dominican Republic, Ecuador, Mexico, Peru	8 (80%)
	Shorter time frames	Argentina, Brazil, Chile, Costa Rica (CCSS), Dominican Republic, Ecuador, Mexico, Panama, Peru	9 (90%)
	Use of protocol models	Chile	1 (10%)
	Decision-making procedures	Argentina, Costa Rica (CCSS), Ecuador, Peru	4 (40%)
	Studies oversight	Argentina, Brazil, Costa Rica (CCSS), Ecuador, Mexico, Peru	6 (60%)
Key ethical issues for review	The use of alternative informed consent processes	Argentina, Brazil, Chile, Colombia, Costa Rica, Peru	6 (60%)
	The collection and store of samples and data for future research	Argentina, Brazil, Chile, Costa Rica, Ecuador, Peru	6 (60%)
	Risk mitigation plans	Argentina, Brazil, Chile, Colombia, Costa Rica, Mexico, Peru	7 (70%)
	Strategies for community engagement in COVID-19 research	Costa Rica	1 (10%)
	Rapid data sharing	Argentina, Costa Rica, Ecuador, Peru	4 (40%)
	Equal access to research benefits	Argentina, Costa Rica, Ecuador, Peru	4 (40%)

All countries' policies called for expedited ethics review of COVID-19 research and 9 of them established specific operational guidelines for this purpose. However, differences were found among countries regarding the number of topics addressed in the guidelines and the level of detail of information given. Argentina, Peru, Brazil, Chile, Ecuador, and Costa Rica issued comprehensive documents that developed 60% or more of the operational guidance categories analyzed. The rest of the countries’ documents included more general statements without precise recommendations for concrete actions for a timely and rigorous review.

Regarding the adoption of key topics for ethics review and oversight, 6 countries have adapted the informed consent process to the context of the pandemic to ensure that potential participants or their legal representatives can make a voluntary decision and to avoid contagion by SARS-CoV-2. Therefore, when it is not possible to obtain informed consent in ordinary ways, alternative mechanisms such as electronic informed consent, use of photographs, phone calls, telemedicine, among others, are allowed to facilitate the process and to support the communication with family members and legal representatives for assistance or proxy consent.

Five countries strengthened the oversight of COVID-19 research to ensure participants' safety, and all of them considered specific operational guidance for RECs regarding this matter. However, no references to the development of REC’s procedures to facilitate the oversight of COVID-19 research considering the rapid production of evidence were found in any country

In 6 countries, the development of mitigation risk plans to protect participants and to prevent the spread of the virus was found as requirements issued by National Regulatory Authorities for both COVID-19 clinical trials and non-COVID 19 clinical trials. In some cases, these plans required ethics review, and in others they only needed to be informed to RECs.

Provisions for the future use of samples and data in research were found in 6 countries' governance frameworks. Five of them considered the use of broad consent processes to facilitate future research, however, only 2 countries (Peru and Ecuador) required Material Transfer Agreements or, at least, their preliminary versions.

General provisions to share research data and results with health authorities and participants were considered in 4 countries. Argentina and Peru contemplated the inclusion of data-sharing plans in the protocol which must be submitted for ethics review, and Ecuador explicitly considered data-sharing to inform public health decision-making.

Four countries contemplated access to benefits from research to participants and their communities. Argentina and Peru required ethics review of these plans or procedures. Costa Rica is the only country in the region that generally recommends engaging communities in research considering their particular social and cultural context. However, dispositions about definitions, specific strategies and plans to engage communities in COVID-19 research were not found in any LA country.

It is worth noting that provisions for public health emergency settings were not found in most of these countries’ pre-pandemic research ethics governance frameworks. Only Brazil, Costa Rica, Panama, and Peru have some disposition for the conduct of research during disease outbreaks. In the case of Panama and Peru, these provisions were issued during the COVID-19 pandemic.

## Discussion

Our findings evidence that several LA countries rapidly issued different instruments for this purpose since the beginning of the pandemic, and these efforts should be recognized. However, the overall lack of emergency ethics preparedness in the region is still worrisome despite the various existing international guidance documents, and the 2018 mandate of PAHO’s Member States to strengthen ethics preparedness for research in emergencies in the region [23]. Furthermore, according to the International Clinical Trials Registry Platform (ICTRP) of WHO [24], all the LA countries included in this review have at least one COVID-19 study registered. Therefore, the call to adjust their research ethics governance frameworks for the conduct of COVID-19 research was a pressing issue for all of them. Below we discuss the main issues that arise from our findings and provide recommendations to strengthen research ethics review during the COVID-19 pandemic and for future health emergencies.

### Organization and coordination

It has been widely discussed the need to evaluate different organizational models to accelerate ethics review during health emergencies [16, 20, 25], yet 4 countries remained under the same ethics review structure. Even though it is not necessary for countries to implement a whole different organization of their ethics review processes, it is of relevance to establish mechanisms for the coordination and communication between research stakeholders, especially considering that these countries host several multicenter studies [3].

In addition, considering the similarities in the legal system and the ethics review processes, countries in the region not exploring organizational alternatives for joint ethics review of COVID-19 research could be losing opportunities to join efforts to define strategies to streamline ethics review beyond their borders during emergency contexts [15, 25, 26].

### RECs “emergency mode” functioning

When general provisions that recommend accelerating ethics review are established in countries’ governance frameworks, they must include specific operational guidance [14, 15, 20]. Otherwise, they turn out to be too vague and leave to the discretion of RECs and their institutions the adjustments of their operating procedures to an “emergency mode”. During emergencies, clear operating procedures will prevent RECs’ difficulties in determining what would be ethically acceptable to streamline in a review process to save valuable time. This will also allow the harmonization of RECs functioning across a country.

Along with operational procedures, rigorous ethics reviews must be ensured so emergency research ethics training for RECs members should be promoted. However, only one country contemplated this topic in its regulation. In emergency contexts, there is a need for increased diligence in the review of research, and special scrutiny is of particular significance when novel, alternative, or complex research designs are proposed [14], in which RECs may not have experience. Alternative research designs, as well as a proliferation of studies—including some incapable of yielding valid results—and the false perception that urgency allows exceptions to high-quality research [4], pose extra challenges to RECs to objectively assess the social value, and scientific validity of research, as well as the risks and benefits for participants. Thus, RECs members need to be trained in order to be sensitive to these ethical challenges in emergency research, and to identify when to call for expert advice if required.

Finally, many countries have increased the oversight and monitoring of COVID-19 research considering the risk–benefit ratio, however specific operating procedures for ethics oversight in light of new scientific evidence were not considered [22]. With controversial cases, such as hydroxychloroquine that came into the spotlight as potential coronavirus treatment, the global research community has witnessed how emerging evidence could impact the scientific validity and social value of ongoing research as well as on participants' safety [27, 28]. Therefore, in emergency settings it is recommended that RECs have procedures to facilitate follow-up and rapid communication between them, researchers, and participants when changes are needed to ensure the ethical acceptability of approved studies.

### Key issues for ethics review during the COVID-19 pandemic

Regarding the informed consent process, the vulnerability and the isolation of COVID-19 patients, precluding any contact with their families or others, have challenged the possibilities of obtaining informed consent in ordinary ways. As discussed in previous outbreaks [7, 8, 10, 11], it is important to highlight that most of the countries were able to rapidly adopt alternative informed consent processes for the pandemic to guarantee it is ethically obtained. This is an important achievement in the region considering that many LA countries have strict laws and regulations regarding the formalities for documenting the process and the decision of research participants [29].

Future use of samples and rapid data sharing were two topics not considered from a comprehensive perspective in most countries. Lessons learned from previous outbreaks have shown the importance of having processes in place to ensure the ethical management of samples and data to allow future research in response to the pandemic, particularly when they are transferred abroad [8, 11, 15, 30]. The use of broad consent processes, proper governance systems to safeguard the interests and well-being of donors, the confidentiality and the quality of the material and data collected, the use of material/data transfer agreements are considered ethical standards [12, 31] that should be subject to the review of a local REC.

Moreover, as in any public health emergency, researchers have the moral obligation to share preliminary data from their research rapidly, even before the publication of research results in scientific journals [11, 32], and as soon as it is quality-controlled for release [17]. During the ongoing pandemic, there has been an explosion of preprints publications (i.e., publications which have not yet passed adequate quality control, e.g., peer-reviewed) in multiple websites to facilitate rapid dissemination of COVID-19 research results among the scientific community. However, as preprints are widely accessed by non-scientific audiences (e.g., the media, general public, policymakers), it has been highlighted the importance of having good preprints publication practices to accurately describe their purpose in order to avoid confusion with peer-reviewed manuscripts and prevent public harm (e.g., public health decision-making, or clinical practice based on unconfirmed results) [33, 34]. Protocols should therefore include plans for rapid dissemination of research data with participants, communities, health authorities, and the scientific community while ensuring scientific integrity standards. RECs could play an important role during ethics review in guiding researchers to disseminate results through platforms that ensure ethics and scientific integrity standards in publications.

No comprehensive provisions were found in any country regarding community engagement strategies as a component of the ethics review of COVID-19 protocols even though international guidelines mention that community engagement strategies should be included as part of study protocols and should be assessed by RECs [12]. Community engagement plans are essential to build and maintain trust and understanding in research activities and to show respect to the affected communities [35, 36], especially in emergency settings characterized by uncertainty and risks of misinformation. As long as these plans help the recruitment of participants, promote the social value of research, and increase the credibility of scientists and their activities to advance COVID-19 research, RECs should require researchers to submit them to ensure ethical research.

Finally, few countries considered provisions on post-trial access or research benefits in their COVID-19 governance frameworks even though fair access to benefits from research is an international ethical standard. During emergency contexts, rapid mechanisms and plans to make available to participants and communities any intervention that proves to be effective, such as agreements between sponsors and health authorities, should be in place in advance and be subject to RECs oversight to avoid exploitation [11, 17, 37]. This was not the case in several LA countries. Despite their participation in multinational COVID-19 vaccine clinical trials, resulting vaccines were not made available to the host countries on a priority basis [38]. The existence of prior agreements (that include fair negotiations terms) could have ensured fair access to the vaccines for host communities.

Despite their relevance in emergency contexts, the above-mentioned topics are weaknesses in the region that existed before the COVID-19 pandemic. In non-pandemic scenarios, biobanking, data sharing, community engagement plans, and post-trial access provisions are not adequately considered in research ethics governance frameworks (see additional file 2), so it was foreseeable that countries would not include them among their COVID-19 regulations. In this context, LA countries should not lose this opportunity for a comprehensive ethics review of COVID-19 research and incorporate these key ethical issues into their regulations. Calling for action on these topics could also lead to further discussion on the necessity of including them into human research governance frameworks beyond the pandemic.

### Preparedness for ethics review in emergency settings

Our results may demonstrate that if not planned in advance, it is difficult for countries to design and implement an adequate research ethics response when a health emergency has already started. Partial and one-time efforts as those adopted by LA countries may not be sufficient to ensure timely and rigorous ethics review of research under challenging circumstances, especially because these circumstances may be evolving and RECs functioning will need adjustments over time. For these reasons, it is crucial to continue insisting LA governments and, in particular, their health authorities, that they need to prepare their countries and strengthen their research capacity to respond timely, efficiently, and ethically to future health emergencies. Planning measures and policies to catalyze ethical research in emergency settings in advance save valuable time and resources, reduce distress, and, ultimately, protect people’s health and lives.

The COVID-19 pandemic has demonstrated that a more comprehensive vision of research ethics during emergencies is needed. Past lessons and the ones we are learning must be the basis from which countries plan and develop their emergency research ethics governance frameworks for the future. To achieve this, further research to explore and analyze countries’ experiences in research and research ethics review in emergency settings, and to develop best practices to ensure ethical research during disease outbreaks in low- and middle-income countries, such as those of LA, should be promoted.

Tailor-made research responses to emergencies have also been part of international organizations that have issued ethics guidance to support countries on research in an emergency-specific context. While this does not mean that context-specific guidance or recommendations are not helpful, it calls for the development of standards and operational guidelines for ethics review and oversight that should be upheld during any public health emergency. International organizations need to work towards the identification of these standards and the most important ethical lessons learned from this and past emergencies around the globe in order to guide countries when planning and developing their emergency research ethics review and oversight policies and strategies.

### Limitations

This study is subject to several limitations. Although we searched legal databases and governmental official websites, we are aware that many documents may not be published online or may be published with delay, particularly because of the times of the pandemic. Some documents may have also been issued after we finished our data collection. Therefore, there might be governmental documents that were not included in this review. Moreover, our search was at a national level, so in federal countries, documents may be issued at a state or province-level but not considered in the study.

## Conclusion

The lack of preparedness for research ethics review and oversight in emergency settings is common among governance frameworks of LA countries. However, as soon as the pandemic was declared, 53% of LA countries rapidly issued legal documents or recommendations to streamline ethics review and oversight of COVID-19-related research. Even though these efforts should be recognized, LA countries tend to adopt partial strategies and operational guidance that may demonstrate a lack of an adequate understanding of emergency research ethics in the region. In this sense, continuing efforts should be directed to strengthen LA countries' research capacity to respond timely and ethically to future health emergencies. For this purpose, past lessons and the ones we are learning from the COVID-19 pandemic should be the basis to develop international standards and operational guidelines for ethics review and oversight during any public health emergency of international concern. It is time to think beyond tailored responses to particular health emergencies because a comprehensive governance framework is needed for the future.

## Supplementary Information

**Additional file 1.** LA Governmental areas contacted.

**Additional file 2.** COVID-19 and general human research ethics governance documents.

## Data Availability

The datasets used and/or analysed during the current study are available from the corresponding author on reasonable request.

## Abbreviations

LA: Latin America
REC: Research Ethics Committee
WHO: World Health Organization
PAHO: Pan American Health Organization
