# Supplementary material for: Governance frameworks for COVID-19 research ethics review and oversight in Latin America: an exploratory study
Source: BMC Med Ethics. 2021 Nov 6;22:147. doi: 10.1186/s12910-021-00715-2 (PMC8571668; doi:10.1186/s12910-021-00715-2)
Supplement: Supplementary file 1 — Additional file 1. LA Governmental areas contacted. [file 12910_2021_715_MOESM1_ESM.docx]

**Additional file 1. Countries and areas of government officials contacted for additional information on the documents found.**

| **Country** | **Area** |
| --- | --- |
| Brazil | National Research Ethics Commission - CONEP |
| Chile | Ministerial Health Research Ethics Commission- CMEIS |
| Costa Rica | National Health Research Council - CONIS |
| Dominican Republic | National Council of Bioethics in Health - CONABIOS |
| Ecuador | Ministry of Public Health |
| Mexico | National Commission on Bioethics - CONBIOETICA |
| Panama | National Committee on Bioethics in Research - CNBI |
