# Supplementary material for: Governance frameworks for COVID-19 research ethics review and oversight in Latin America: an exploratory study
Source: BMC Med Ethics. 2021 Nov 6;22:147. doi: 10.1186/s12910-021-00715-2 (PMC8571668; doi:10.1186/s12910-021-00715-2)
Supplement: Supplementary file 2 — Additional file 2. COVID-19 and general human research ethics governance documents. [file 12910_2021_715_MOESM2_ESM.docx]

**Additional file 2. Identified governmental documents on COVID-19 research ethics review in LA countries, and general human research ethics governance framework.**

| **Country** | **COVID-19 research ethics review and oversight framework** | **Human research ethics governance framework** |
| --- | --- | --- |
| **ARGENTINA** | Resolution 908/2020 of the Ministry of Health: Ethical and operational guidelines for accelerated ethics review of COVID-19-related human research <http://servicios.infoleg.gob.ar/infolegInternet/anexos/335000-339999/337359/norma.htm>  Communication ANMAT, May 2020. <https://www.argentina.gob.ar/noticias/medidas-y-recomendaciones-en-los-estudios-de-farmacologia-clinica-durante-la-pandemia-covid> | Resolution 1480/2011 of the Ministry of Health: Guidelines for Human Health Research <http://servicios.infoleg.gob.ar/infolegInternet/anexos/185000-189999/187206/norma.htm>  Disposition 6677/2010 of ANMAT: Good clinical practices for pharmacological studies <http://www.anmat.gov.ar/comunicados/dispo_6677-10.pdf> |
| **PERU** | PNIH Chief Resolution Nº 096-2020-J-OPE/INS that creates the National Transitory Research Ethics Committee for the ethics review and oversight of COVID-19 clinical trials.  [RJ_096-2020-J-OPE-INS20200727-24078-s7qgfw.pdf (](https://cdn.www.gob.pe/uploads/document/file/1049675/RJ_096-2020-J-OPE-INS20200727-24078-s7qgfw.pdf)[www.gob.pe](http://www.gob.pe)[)](https://cdn.www.gob.pe/uploads/document/file/1049675/RJ_096-2020-J-OPE-INS20200727-24078-s7qgfw.pdf)  PNIH Chief Resolution Nº 097-2020-J-OPE/INS that approves the procedure for the ethics review of COVID-19 clinical trials.  [RJ_097-2020-J-OPE-INS20200727-24078-4mz72l.pdf (www.gob.pe)](https://cdn.www.gob.pe/uploads/document/file/1049676/RJ_097-2020-J-OPE-INS20200727-24078-4mz72l.pdf)  Directoral Resolution Nº 120-2020-OGITT/INS that approves the NTREC-COVID19 operating procedures (April, 2020)  <https://ensayosclinicos-repec.ins.gob.pe/regulacion/normatividad-vigente/314-investigaciones-con-covid-19>  PNIH Chief Resolution Nº 139-2020-J-OPE/INS that approves guidelines for the conduct of clinical trials during the COVID-19 pandemic.  [RJ_Nº_139-2020Doc.Tec.-Pautas_Ejec.Ensay.Clinicos20200727-24078-m50hft.pdf (www.gob.pe)](https://cdn.www.gob.pe/uploads/document/file/1049749/RJ_N%C2%BA_139-2020Doc.Tec.-Pautas_Ejec.Ensay.Clinicos20200727-24078-m50hft.pdf)  Supreme Decree Nº 014-2020-SA that establishes measures for the adequate conduct of COVID-19 clinical trials in the context of the health emergency  <https://www.gob.pe/institucion/minsa/normas-legales/483022-014-2020-minsa> | Supreme Decree Nº 021-2017-SA that approves the Clinical Trials Regulations. [El Peruano - Aprueban Reglamento de Ensayos Clínicos - DECRETO SUPREMO - N° 021-2017-SA - PODER EJECUTIVO - SALUD](https://busquedas.elperuano.pe/normaslegales/aprueban-reglamento-de-ensayos-clinicos-decreto-supremo-n-021-2017-sa-1538902-2/)  Technical document: Ethical considerations for health research involving human beings, approved by Ministerial Resolution Nº 233-2020-MINSA.  [RM_233-2020-MINSA_Y_ANEXOS.PDF (www.gob.pe)](https://cdn.www.gob.pe/uploads/document/file/662949/RM_233-2020-MINSA_Y_ANEXOS.PDF) |
| **ECUADOR** | Ministerial Agreement Nº 0003, Regulation for health research during the health emergency (repealed).  <https://www.registroficial.gob.ec>  Ministerial Agreement Nº 00104, Regulation for the approval and the conduct of health research related to COVID-19.  <https://www.registroficial.gob.ec> | Ministerial Agreement Nº 4889, Human Research Ethics Committees Regulations.  [https://www.gob.ec/sites/default/files/regulations/2018-10/Documento_Reglamento%20Comités%20Ética%20Investigación%20Seres%20Humanos.pdf](https://www.gob.ec/sites/default/files/regulations/2018-10/Documento_Reglamento%20Comit%C3%A9s%20%C3%89tica%20Investigaci%C3%B3n%20Seres%20Humanos.pdf) |
| **PANAMA** | Resolution Nº 373.  [Gaceta Oficial Digital (b-cdn.net)](http://minsa.b-cdn.net/sites/default/files/normatividad/resolucion_ndeg373_de_13_de_abril_de_2020_gaceta_29005_procedimiento_acelerado_ips_en_edb.pdf) | Law Nº 84.  [Gaceta Oficial Digital (b-cdn.net)](http://minsa.b-cdn.net/sites/default/files/normatividad/ley_84.pdf)  Operating procedures of the National Committee of Bioethics in Research.  <https://cnbi.senacyt.gob.pa/procedimientos-operativos-del-cnbi/> |
| **COSTA RICA** | Communique 1: Specific considerations for biomedical research in the framework of the COVID-19 pandemic.  Communique 2: Recommendations for the conduct of biomedical research during the health emergency in Costa Rica.  [comunicado_cec_oac_oic_20082020.pdf (ministeriodesalud.go.cr)](https://www.ministeriodesalud.go.cr/gestores_en_salud/conis/circulares/comunicado_cec_oac_oic_20082020.pdf)  Manual of procedures to streamline ethics review and oversight of biomedical COVID-19 research of the CCSS  <https://www.cendeisss.sa.cr/wp/wp-content/uploads/2021/01/Manual-procedimientos-revision-expedita-17.07.2020.pdf> | Law 9.234 on Biomedical Research  [Sistema Costarricense de Información Jurídica (pgrweb.go.cr)](http://www.pgrweb.go.cr/scij/Busqueda/Normativa/Normas/nrm_texto_completo.aspx?param1=NRTC&nValor1=1&nValor2=77070&nValor3=122799&strTipM=TC)  Executive Decree Nº 39061-S (2015, update 2016) that regulates the Law on Biomedical Research  [Sistema Costarricense de Información Jurídica (pgrweb.go.cr)](http://www.pgrweb.go.cr/scij/Busqueda/Normativa/Normas/nrm_texto_completo.aspx?param1=NRTC&nValor1=1&nValor2=79779&nValor3=103451&strTipM=TC) |
| **BRAZIL** | ORIENTAÇÕES PARA CONDUÇÃO DE PESQUISAS E ATIVIDADE DOS CEP DURANTE A PANDEMIA PROVOCADA PELO CORONAVÍRUS SARS-COV-2 (COVID-19)  [Comissão Nacional de Ética em Pesquisa](http://www.fo.usp.br/wp-content/uploads/2020/07/Orienta%C3%A7%C3%B5es-condu%C3%A7%C3%A3o-de-pesquisas-e-atividades-CEP.pdf)  Technical Note 23/2020  [Anvisa](http://antigo.anvisa.gov.br/documents/219201/5923491/NOTA+T%C3%89CNICA+N%C2%BA+23-2020+-+GGMED.pdf/fc71b725-0457-43d3-aacc-4bceb3f4127f) | Resolution 466/12 of National Health Council.  [Conselho Nacional de Saúde](https://conselho.saude.gov.br/resolucoes/2012/466_espanhol.pdf)  Operational norm N° 001/2013 of National Health Council  [Ministério da Saúde](http://www.hgb.rj.saude.gov.br/ceap/Norma_Operacional_001-2013.pdf)  Resolution n° 446/11 of National Health Council  [Ministério da Saúde](http://bvsms.saude.gov.br/bvs/saudelegis/cns/2011/res0446_11_08_2011.html)  Resolution 506-2016  [Conselho Nacional de Saúde](https://conselho.saude.gov.br/resolucoes/2016/Reso_506.pdf)  Resolution 580-2018  [Conselho Nacional de Saúde](https://conselho.saude.gov.br/resolucoes/2018/Reso580.pdf) |
| **MEXICO** | BIOETHICS IN THE FACE OF THE COVID-19 PANDEMIC  <https://www.gob.mx/cms/uploads/attachment/file/544217/BIOETHICS_IN_THE_FACE_OF_THE_COVID-19_PANDEMIC.pdf>  Extraordinary Measures in relation to Clinical trials during the pandemic  <https://www.gob.mx/cofepris/es/articulos/medidas-extraordinarias-en-relacion-a-estudios-clinicos-ante-la-pandemia-de-covid-19?idiom=es>  Communique N° 007 COVID-19  <https://www.gob.mx/cms/uploads/attachment/file/555510/Suspension_y_funcionamiento_CEI.pdf> | General Health Law  <http://dof.gob.mx/nota_detalle.php?codigo=4652777&fecha=07/02/1984#:~:text=%2DLa%20presente%20Ley%20reglamenta%20el,Federaci%C3%B3n%20y%20las%20entidades%20federativas>  Regulation of the Law in Aspects of Health Research  <http://www.diputados.gob.mx/LeyesBiblio/regley/Reg_LGS_MIS.pdf>  National Guideline for the integration and functioning of RECs  <https://www.uv.mx/ics/files/2019/04/Gui_a_CEI_2018_6ta_Edicion.pdf> |
| **CHILE** | Recommendations for scientific ethics committees for research protocols review in the context of the COVID-19 pandemic of the CMEIS  <https://www.uta.cl/wp-content/uploads/2020/06/ProtocoloCECc.pdf> | Law 20.120 Scientific Human Investigation, it’s genome and prohibition of human clonations  [Ley-20120 22-SEP-2006 MINISTERIO DE SALUD, SUBSECRETARÍA DE SALUD PÚBLICA - Ley Chile - Biblioteca del Congreso Nacional (bcn.cl)](https://www.bcn.cl/leychile/navegar?idNorma=253478)  Decree 114-2011  [Decreto-114 19-NOV-2011 MINISTERIO DE SALUD, SUBSECRETARÍA DE SALUD PÚBLICA - Ley Chile - Biblioteca del Congreso Nacional (bcn.cl)](https://www.bcn.cl/leychile/navegar?idNorma=1032919&idParte=9206067&idVersion=)  Exempt Resolution 403-2011  <https://www.ispch.cl/sites/default/files/normativa_anamed/ensayos_clinicos/Resoluci%C3%B3n%20Exenta%20403.pdf>  Exempt Resolution 183-2016  [Resolución-183 EXENTA 22-MAR-2016 MINISTERIO DE SALUD - Ley Chile - Biblioteca del Congreso Nacional (bcn.cl)](https://www.bcn.cl/leychile/navegar?idNorma=1088598) |
| **COLOMBIA** | External Circular 1000-174-20, INVIMA  <https://www.invima.gov.co/medidas-y-acciones-excepcionales-aplicables-al-desarrollo-de-ensayos-clinicos-durante-la-vigencia-de-la-emergencia-por-covid-19> | Resolution 8430 of 1993, Ministry of Health.  <https://www.minsalud.gov.co/sites/rid/Lists/BibliotecaDigital/RIDE/DE/DIJ/RESOLUCION-8430-DE-1993.PDF>  Resolution 2378 of 2008, Ministry of Health  <https://www.invima.gov.co/normatividad-interna/-/document_library/CMKVmmc4XvpT/view_file/1024889> |
| **DOMINICAN REPUBLIC** | Communiquee: The CONABIOS in times of COVID-19  <http://conabios.gob.do/wp-content/uploads/2020/04/comunicado-covid-19.pdf> | General Health Law N°42-01 <https://www.semma.gob.do/media/1704/ley-general-de-salud.pdf>  Resolution 12-2008  <http://conabios.gob.do/wp-content/uploads/2019/12/Resolucio%CC%81n-Conabios.pdf> |
